# Lamellar body count as a diagnostic test in predicting neonatal respiratory distress syndrome

**DOI:** 10.3325/cmj.2012.53.234

**Published:** 2012-06

**Authors:** Tea Štimac, Oleg Petrović, Robert Krajina, Mirko Prodan, Lidija Bilić-Zulle

**Affiliations:** 1 Department of Gynecology and Obstetrics, Perinatology Unit, University Hospital Rijeka, Rijeka, Croatia; 2 Clinical Department of Laboratory Diagnosis, University Hospital Center Rijeka and Department of Medical Informatics, Rijeka University School of Medicine, Rijeka, Croatia

## Abstract

**Aim:**

To determine the lamellar body count (LBC) cutoff value for fetal lung maturity and to evaluate the clinical usefulness of LBC in predicting the severity of neonatal respiratory distress syndrome (RDS).

**Methods:**

A prospective study was conducted from 2002 until 2010. LBC was estimated in uncentrifugated amniotic fluid samples using Cell-Dyn 1800 analyzer. Amniotic fluid samples were obtained by amniocentesis or by puncturing embryonic membranes during cesarean section. The presence of mild, moderate, and severe RDS was assessed by neonatologist.

**Results:**

A total of 313 patients with singleton pregnancies (24-41 weeks) were included in the study and 294 met the inclusion criteria. RDS was diagnosed in 28 neonates – mild in 8, moderate in 10, and severe in 10. In premature neonates (<37 gestational weeks), significant differences in LBC were only found between the subgroup without RDS and the group with moderate and the group with severe RDS (*P* < 0.001). In all neonates, significant differences were found between neonates without RDS and neonates with RDS. Using LBC cutoff value of ≥20,000/µL, sensitivity, specificity, and positive and negative predictive values of LBC in determining mature fetal lungs were 96%, 88%, 45.6%, and 99.5%, respectively.

**Conclusion:**

This study suggests that LBC cutoff value of ≥20,000/µL can predict pulmonary maturity and reduce the risk of neonatal respiratory distress syndrome.

Respiratory distress syndrome (RDS) occurs in infants delivered before the completion of fetal lung maturation and remains a major cause of neonatal morbidity and mortality in premature infants (1). RDS affects approximately 1% of all live births, but 10 to 15% of all infants with a birth weight less than 2500 g (2). The risk of RDS is increased with prematurity, but is also significant in infants born by electively scheduled cesarean delivery between 38 and 39 weeks of gestation (3). Therefore, the American College of Obstetricians and Gynecologists recommends the documentation of fetal lung maturity for elective deliveries at less than 39 weeks of gestation to avoid neonatal RDS (4). There are several antenatal tests for lung maturity. Evaluation is commonly based on laboratory measurements of the concentrations of particular components of pulmonary surfactant (4-7). Most of them are either complex, expensive, or with low diagnostic efficiency (8). Amniotic fluid lamellar body count (LBC) is one among various biophysical tests, based on measuring the concentration of pulmonary surfactant in amniotic fluid (9).

Lamellar bodies (LB) are storage form of surfactant within type II pneumocytes and are actively secreted into the alveolar space and hence into the amniotic fluid (4). The size of LBs, similar to platelets, permits the use of a standard hematologic counter to quantify LB concentration in amniotic fluid samples. This technique quantitatively estimates surfactant production and thus can predict the degree of fetal lung maturation. Still, there are no clearly established protocols and cutoff values for LBC that predict RDS (4). The maturity thresholds for LB vary due to different factors, ie, centrifugation (leads to substantial loss of the LBs), sample contamination (meconium or platelets from the blood can be counted as LBs), and sample type (vaginal pool specimen containing mucus can artificially increase the LBC) (10,11). Depending on the protocols, the cutoff values of LBC that predict the absence of neonatal RDS can vary from 15 000 to even 80 000/µL (4,10,11). Also, the type of particle count analyzer used is critical in setting the cutoff values, so it has been proposed to establish analyzer-specific references for LBC (1). The aim of our investigation was to evaluate the LBC cutoff value using Cell-Dyn 1800 analyzer (Abbott Diagnostics, Abbott Park, IL, USA) and evaluate the efficiency in predicting different grades of neonatal RDS.

## Materials and methods

A prospective study was conducted from the beginning of 2002 until the end of 2010 at the Department of Perinatology, University Hospital Rijeka. Amniotic fluid samples were obtained from women with singleton pregnancies (24-41 gestational weeks) who underwent transabdominal amniocentesis under ultrasound control for fetal lung maturity determination because of inevitable delivery and from those who underwent elective cesarean deliveries by puncturing visible embryonic membranes. In a 9-year period, 26 473 deliveries were performed at our institution. We excluded those with gestational pathology that can influence fetal lung maturity (cases with premature rupture of membranes, oligohydramnios/hydramnios, and infants with major congenital or chromosomal abnormalities). A total of 313 women met the study criteria, agreed to participate in the study, and provided written consent prior to the amniocentesis. The ethical approval was provided by ethics committees of the University Hospital Rijeka and University of Rijeka School of Medicine. Out of 313 women, 19 were excluded because the deliveries occurred more than 7 days after amniocentesis or were done in other institutions so medical charts from the delivery were not available.

Gestational age was calculated from the last menstrual period and confirmed by ultrasound measurement during the first 20 weeks of pregnancy. Samples containing 5 mL of amniotic fluid were immediately transported to the clinical laboratory in a capped plastic syringe and analyzed according to an established protocol (10,12). LBC was estimated in uncentrifugated amniotic fluid samples using the Cell-Dyn 1800 analyzer and its platelet channel. All samples containing blood or meconium were rejected.

The neonatal respiratory status and existence of RDS were reviewed by attending neonatologists, who had not been informed about the concentration of LBs. All neonates were divided into four groups according to clinical symptoms of RDS and therapy measures: group A – without symptoms, group B – mild RDS (expiratory grunting, transitional tachypnea, O_2_ in therapy), group C – moderate RDS (prior symptoms + bluish color of the skin, chest wall retraction, in therapy O_2_>40% and/or continuous positive airway pressure, sometimes surfactant), and group D – severe RDS (prior symptoms + cyanosis, apnea, therapy includes all intensive supportive measures, surfactant, mechanical ventilation). Diagnosis of moderate and severe RDS was confirmed by findings on chest radiographs. The special subgroup of premature deliveries (<37 weeks) was extracted from the group A with a purpose to compare this group with the groups B, C, and D.

Statistical analysis was performed using MedCalc Software (Mariakerke, Belgium), version 11.3.3.0. We used χ^2^ test for values assessed on a nominal scale and Kruskal-Wallis ANOVA for non-normally distributed data. Mann-Whitney U test was used for subsequent comparison between different groups. The cutoff value for predicting a high likelihood of RDS was derived from the receiver-operating characteristic (ROC) curve. We also determined Spearman rank correlation coefficient between LBC and gestational age and calculated sensitivity, specificity, positive predictive value, and negative predictive value for LBC in predicting fetal lung maturity. Also, the likelihood ratios for positive (LR+) and negative tests (LR-) were determined. For all tests, a value of *P* < 0.05 was considered significant.

## Results

**Figure 1:**
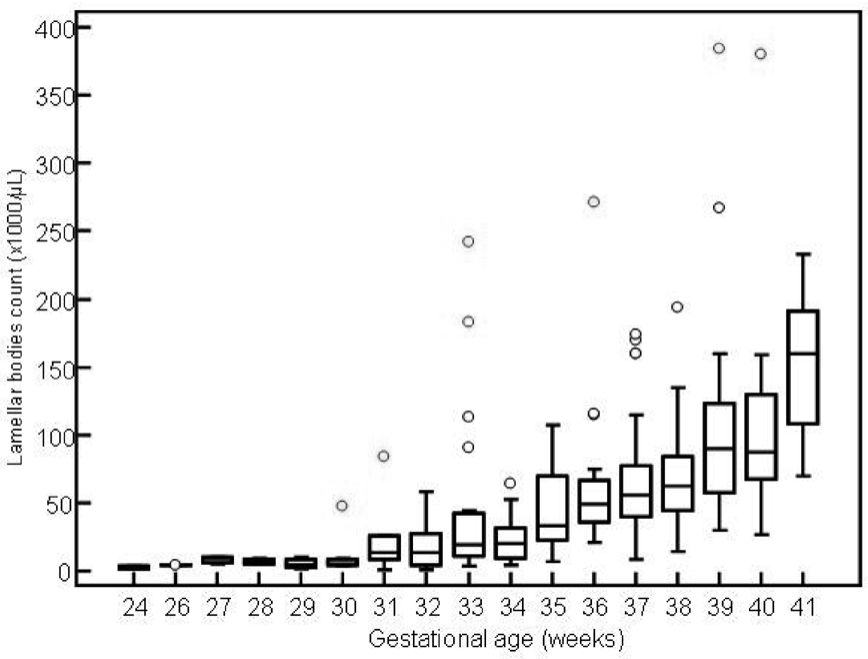
Median values and range of lamellar body counts in amniotic fluid according to gestational age (n = 294).

**Figure 2:**
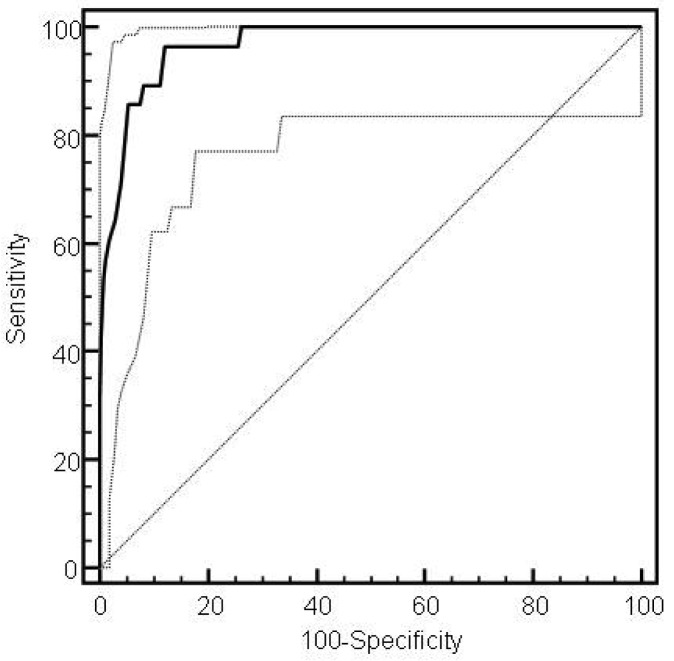
Receiver operating characteristic curve for lamellar body count in predicting neonatal respiratory distress syndrome.

Nineteen out of 313 women were excluded either because their deliveries occurred more than 7 days after amniocentesis or their medical charts were not available. Among 294 patients who met the study criteria, there were 43% nuliparous women. Median maternal age was 30 years (range 16-45) and median gestational age was 37 weeks (range 24-41). Most of them (73%) were delivered by cesarean section. Advanced gestational age was associated with higher LB concentration (Spearman rank correlation coefficient; r = 0.600, *P* < 0.001) (Figure 1). A total of 47% of neonates were female (n = 142), median weight was 2585 g (range 550-5620), and 13 out of 294 had 5-minute Apgar score <7. RDS was diagnosed in 28 (9.5%) neonates and all of them were premature – 8 neonates had mild, 10 had moderate, and 10 had severe RDS. All 3 neonatal deaths due to acute RDS were in the group of neonates with the most severe clinical form of RDS (24 weeks – LBC 3000/µL; 24 weeks – LBC 2000/µL; 31 weeks – LBC 1000/µL). We found significant differences between neonates with (group B, C, and D) and without RDS (group A) in gestational age, birth weight, 5-minute Apgar score, and mode of delivery (Table 1). We also found significant differences in LBC between the neonates without RDS and all three groups of neonates with various severities of RDS, but there were no significant differences between the groups with mild, moderate, and severe neonatal RDS (Table 2). In premature deliveries <37 gestational weeks, significant differences in amniotic fluid LBC were only found between the subgroup without RDS and the groups with moderate and severe RDS (*P* < 0.001). The area under the ROC curve for LBC in RDS prediction in neonates was 0.97 (95% confidence interval, 0.94-0.99) (Figure 2). The sensitivity, specificity, and positive and negative predictive values for LBC value ≥20,000/µL in predicting fetal lung maturity were 96%, 88%, 45.6%, and 99.5%, respectively. The likelihood ratio for a positive test was 8.02 and for a negative test was 0.04.

**Table 1:** Characteristics of neonates with (n = 28) and without respiratory distress syndrome (RDS) (n = 266)

Characteristics	Without RDS	With RDS	*P*
Gestational age in weeks	37 (30-41)*	30 (24-37)*	<0.001^†^
Birth weight in grams	2690 (660-5620)*	1240 (550-3580)*	<0.001^†^
5-min Apgar score <7 (n, %)	24 (9)	20 (71)	<0.001^‡^
Female sex (n, %)	127 (47)	15 (53)	0.698^‡^
Cesarean section (n, %)	188 (70)	26 (92)	0.022^‡^

*Median (range).

†Mann-Whitney test.

‡χ^2^ test.

**Table 2:** Amniotic fluid lamellar body counts according to the presence and severity of neonatal respiratory distress syndrome (RDS)

RDS (number of cases)	LBC × 1000/µL median (range)	Gestational age median (range)
Group A (n = 266)	55.5 (4-384)*	37 (30-41)
Without, gestation <37 weeks (n = 99)	26 (2-271)^†^	35 (30-36)
Group B (n = 8)	12 (3-33)	32.5 (30-36)
Group C (n = 10)	5.5 (1-10)	28 (27-33)
Group D (n = 10)	3.5 (1-8)	29.5 (24-32)

*Significant differences in amniotic lamellar body counts (LBC) between the group A and other three groups of neonates with RDS.

†Significant differences in amniotic LBC between the subgroup of premature neonates (<37 weeks) without RDS and the groups C and D.

## Discussion

The cutoff value for LBC in predicting fetal lung maturity in our study was ≥20,000/µL. Because of different types of count analyzer and various protocols, different authors propose different cutoff values (13-15). At our institution, we established a protocol for LBC testing using Cell-Dyn 1800 automatic analyzer. We did not collect amniotic fluid samples from the vagina and samples were free of blood and meconium, and more importantly, not centrifugated before the analysis, because this can considerably decrease LBC (6).

We demonstrated that advancing gestational age led to higher LB concentrations, with significant differences in amniotic fluid LBC between healthy neonates and those with RDS. This finding confirms gestational age as an independent factor with important influence on amniotic fluid LBCs. The group of neonates with RDS had significantly lower gestational age, birth weight, and 5-minute Apgar score than healthy neonates. Significant differences in LBCs were found between the neonates without RDS and all three groups of neonates with various severities of RDS, but there were no significant differences between the groups with mild, moderate, and severe RDS. Because the neonates with RDS were all premature, we wanted to compare them with a subgroup of premature newborns derived from the group of healthy neonates without RDS (group A). The antenatal amniotic fluid LBC method was able to differentiate between the neonates without RDS and the neonates who will develop moderate and/or severe forms of acute RDS. However, based on our results that more severe forms of RDS were accompanied by lower median LBC, but with no significant differences in LBC between the three groups with RDS, we can only hypothesize that amniotic fluid LBC cannot differentiate between moderate and severe forms of RDS. Such result may be a consequence of a relatively small number of cases with mild, moderate, and severe RDS. We are convinced that clinically significant result of LBC≥20,000/µL of amniotic fluid as a cutoff value can predict pulmonary maturity with high sensitivity and specificity and thus reduce the possibility of moderate and severe RDS. The calculated LBC cutoff value was lower than the majority of reported cutoff values in literature, probably because we classified acute neonatal RDS in three grades according to severity (4,8). LBC cutoff value of ≥20,000/µL can predict pulmonary maturity and reduce the risk of the neonatal RDS. Amniotic fluid LBC performed on cell-counting equipment available in most clinical laboratories is a simple, rapid, inexpensive, and the most practical antenatal method for the efficient evaluation of fetal lung maturity and prediction of neonatal RDS.
